# Cocain Pressure Anesthesia—Its Dangers and Failures

**Published:** 1905-11-15

**Authors:** E. T. Loeffler


### COCAIN PRESSURE ANESTHESIA—ITS DANGERS AND FAILURES.

BY E. T. LOEFFLER, B.S., D.D.S.

Read before the Michigan Dental Association, July 10, 1905.

For a number of years the dental as well as other medical literature has teemed with testimony to the wonderful anesthetic effects of the hydrochlorate of cocain. Under ordinary circumstances we are inclined to wait for the elapse of some time before granting the truth of such extraordinary claims, as are commonly made in behalf of a new remedy.

The available supply of the salt has thus far continued to be somewhat limited and expensive, and yet the amount has been sufficient to establish its marvelous power. We are amply justified, it seems to me, in proclaiming the announcement of this fact to be the most important discovery made in therapeutics since Morton astonished the world with his demonstration of the anesthetic power of ether.

No doubt, much yet remains to be done in the way of experiment and observation before the precise sphere of this drug as a dental anesthetic can be defined. Pressure anesthesia is still in its infancy, and the conservative practitioner naturally has an aversion for new remedies and methods. This method, if successful, enables us to perform one of the most delicate operations without pain, and renders possible the immediate filling of a carious tooth without removal of the pulp, therefore doing away with the tedious, painful and doubtful filling of root canals and their treatment. This is a fact much desired by every operator, to be able to practice painless dentistry without danger to the pulp.

Dangers.—The careless manner in which this drug has been used by the medical and dental professions can be attributed only to ignorance of its toxic character. The evidence to prove cocain a poison is now so ample, that no excuse will avail to exonorate the doctor who, not heeding the lesson taught by the gruesome record, fails to use it with the care its toxic energy demands. It is a remedy peerless for good in certain conditions, but its power for ill must never be lost sight of, if one would conserve the best interest of those on whom it may seem wise to use it.

To rob an operation entirely of pain, a claim based upon a few week's trial and too often without specific directions or care as to the amount used, or making any allowance for differences in age, sex or constitutional susceptibility, is a claim that must be carefully made.

While constitutional disturbances are a possibility, the principal dangers in the use of cocain by this method are those affecting pulp and peridental membrane. The most remarkable feature about the alkaloid, cocain is the great variability of its action, and it is to an ignorance of the knowledge of this fact, probably, that accidents and failures are due. One author gives a history of thirteen deaths out of 250 cases of poisoning arising from the medicinal use of cocain.

Cocain belongs to a class of toxic substances, known as nerve poisons, the most important of which are, chloral, opium and its alkaloids, cocain, atropine, hyoscyamine, daturine, nicotine, conine, cicutoxine, strychnine, cytisine and curarine.

As a matter of comparison it may be interesting to give the action of cocain as held by different anthorities. Warthin claims that it produces peripherally, a dulling of the excitability of the sensory nerve endings; centrally, first a stimulation and later a paralysis. The chronic use of the drug gives rise to symptoms similar to those of chronic morphinism. Ziegler claims that nerve poisons are characterized chiefly by the fact that, in spite of the severity of the symptoms, as shown in the form of irritations and paralysis, anatomical changes either cannot be recognized at all, or are confined to structural changes in the proto-plasm of inidvidual nerve cells which are similar in character to those noticed from other poisons. This is especially the case when the poison is quickly fatal, while if the poisoning runs a protracted course, or in the case of chronic poisoning from small doses, extending over months and years, there are very often found marked anatomical changes manifested in the form or degenerations. Kobert, one of the greatest authorities in the classification of poisons, states that the organic bases (strychnine, cocain, atropine, etc.), have an unexplained action.

Hare claims that coca and its alkaloid, when taken internally, produces a sense of exhilaration and pleasure. Locally it causes a blanching followed by a marked congestion; also that it acts as a heart and respiratory stimulant, but only in large doses.

Mosso believes that decreased sensation is due to its action on the spinal cord, and in case of a poisonous dose, we have convulsions of a cerebral origin and are both clonic and tetanic in type. According to Reichert, there is an extraordinary rise in temperature due to increase of heat production. In small doses it has no effect, however.

Koller, of Vienna, who was the first to demonstrate the use of cocain as a local anesthetic in operations on the eye, asserts that it produces its effect by constricting the blood vessels of the iris; opthalmologists, however, generally assert that it causes dilatation by stimulating the peripheral ends of the sympathetic nerve. Some authors claim that it has a selective action as shown by its action on the sense of taste and smell. Cushney says that a good deal of discussion has arisen as to whether the action on the sensory termination is a specific one or not, and he concludes by stating that we must consider cocain as a general proto-plasm poison with some selective power for certain nerve tissues, but with no specific affinity for sensory rather than for motor terminations. He further claims that it produces besides a loss of sensation, a feeling of constriction and a distinct palor and contraction of the vessels.

According to very recent and careful researches made by Parke, Davis & Co., cocain does not possess a selective action and is not an astringent. The arterial pressure is increased, but the increase is probably due, first, to a stimulation of the vaso-motor, centre in the medulla; and, second, to a stimulation of the heart. The increased action upon the heart is probably due to a depressing influence on the cardio-inhibatory apparatus, both centric and peripheral, causing an acceleration of the pulse. Cocain is a cerebral stimulant, producing peculiar mental excitement, ending after large doses in narcosis with epileptiform convulsions which are probably of central origin.

Upon striated muscle it appears to have a very peculiar though often a very fatal action, which is not manifested during the poisoning. It has been asserted that cocain acts as a powerful diuretis, but the drift of present evidence is to show that it has no definite influence upon the amount of urine secreted. Dropped into the eye, cocain occasionally dilates the pupil without increasing the intraocular pressure, or completely paralysing the accommodations. It is a powerful stimulant of the respiratory centres, increasing the rapidity and fullness of the respiration, but in doses severely large it, for a time, causes the respiration to become very shallow and finally paralyses the centres. Moderate doses are said to increase and large doses to paralyze peristalsis.

In cocain poisoning there is at first increased reflex activity which is chiefly due to a distinct action upon the spinal cord, the sensory side of the cord being probably more sensative to the drug than the motor side. Toxic doses depress and finally paralyze the sensory nerves and in a much less degree those which carry motor stimuli.

The occasional over-effects of small doses are quite remarkable. Thus four drops of a 2 percent solution in the eye, produced in an old lady intoxication which persisted for four days; eight drops of a 10 percent solution in the eye of a girl 12 years old, caused violent poisoning, and even one drop of a 1 percent solution in a child fourteen years of age is said to have been followed by very severe symptoms. A number of cases are on record in which one grain of the alkaloid given hypodermically has caused a severe fainting spell. Death is reported in several cases, twelve drops of a 4 percent solution given hypodermically to a girl of eleven years caused death in forty seconds.

So we can readily see that the exact manner in which cocain acts is not definitely known. It evidently produces its effects as all poisons do, because of its chemical nature.

One eminent authority has said that scientific research is, perhaps, the most important factor for opening up to everyone the possibility of using drugs or remedies with a definite idea of the reason for their employment. From a careful study upon the subject of therapeutics, many text books fail to make clear the close relationship which ought to exist between the results reached by the pharmacologist on the one hand and the clinician on the other. Furthermore, it seems that, in some cases at least, a special effort has been made to divorce these two branches by a prolonged mental effort in some other direction.

It is true that, in special cases, science and practice seem to be diametrically opposed and only further research will be able to clear up the apparent contradiction.

An intelligent use of cocain hydrochlorate cannot be brought about unless we obtain a more exact knowledge of its pharmacology and the relationship which it bears to its practical application. One reason why we fail, even when injecting cocain into dentin is that we do not know its proper reaction.

Another very good reason for our failures is due to the fact that we do not thoroughly understand the nature of the tissues into which we inject it. There is evidently a great lack of uniformity in structure.

According to Huber the nerve fibres extend only to the odontoblastic layer. The exact nature of the contents of the tubuli is not definitely known, although is supposed to be of the nature of protoplasm. Every variation in structure would, it seems to me, necessitate a variation in the method of application.

A third reason for failures in pressure anesthesia, perhaps, is due to the peculiar chemical nature of the drug. Upon heating it is changed into three distinct compounds, viz: Methyl, alcohol, $CH_4O$; Benzoic Acid, $C_7H_6O_2$, and Ecgonine, $C_0H_{15}NO_3$, the latter having a sweetish bitter taste and soluable in water but not in alcohol. Ecgonine possesses entirely different powers from cocain, and it is claimed by some authors that this compound is responsible for some of the evil effects and failures which have resulted from badly prepared cocain.

A fourth reason why cocain under pressure fails is on account of imperfect instruments. The pressure cannot be controlled or maintained. The operator is handicapped in applying the necessary amount of pressure and the patient is at times subjected to a great deal of unecessary pain and annoyance. The ideal appliances would be one in which the pressure could be applied evenly, and if possible contain some device for recording the amount of pressure.

Such an appliance, I am told, has been invented by Dr. Price, of Cleveland.

Peculiar shapes of instruments and lack of familiarity are responsible for a large share of the failures in applying this process. There is absolutely nothing so much to be desired by an operator as to be able to practice painless dentistry without danger to the pulp. It matters not so much which form of instrument is to be used, because, if the method proves to be a success, there will very soon be on the market instruments more satisfactory and easier of manipulation.

There seems to be no doubt in the minds of prominent authorities that anesthesia can be produced by this method. Experiments are being carried on by different societies to determine its effect on the dental pulp. Experiments with the same object in view have been carried on in the clinic of the dental department of the University of Michigan during the last session under the writers instructions but no definite report can be submitted at this time.

As a caution against the use of strong solutions, I beg to call your attention to the experiences of Dr. Maurel, of Tollouse, who, a few years ago, carried on a series of experiments with reference to the toxic effects or properties of cocain, clearly demonstrating the advisability of using weak solutions. He found that the lucocytes became rigid and spherical, increased in size and lost their adhesion to the walls of the vessels. That the capillary vessels contracted, a circumstance which often gives rise to thrombosis and embolism especially to pulmonary emboli. He further stated that these changes in the leucocytes take place also with small doses of cocain when the latter is present in considerable proportion, 10 percent, for example, a fact which explains the serious ill effects sometimes following such employment.

Pulmonary embolism being the most serious effect of cocain intoxication it seems plausible that intra-arterial injections in the direction of important viscus would be much less dangerous than intravenous injection.

The author's experiment show that this theory is well founded, seeing that he was able to inject into the femoral artery of a rabbit 10 percent of cocain for each gram of the animal's weight without killing it. The toxic effects of cocain is not restricted to its action on the leucocytes, but exerts its influence also in other directions, the most notable of these effects being contraction of the smaller vessels.

When we apply pressure the hydraulic principle is reversed. We get a small pressure at the small end because this pressure is on a large surface and takes effect only at the small point. To overcome this, we increase the pressure by applying the principle of leverage by means of a forcep handle. The more nearly the handles are closed the greater is the force. One thing about this is that it is difficult to hold in position; your hand almost becomes paralyzed. All those with whom I have had conversation, say that the question of getting the contact and keeping it, is the difficult point.

Some of the instruments on the market are provided with a kind of tension spring. The principle here is when you get the pressure it will be maintained by the coil spring. The important feature in this case is that when you get a certain amount of pressure, and the patient can generally tell you when there is enough, you can hold it there and don't have to cramp your hand. If we had a means of adjusting this pressure by working the finger or thumb in the direction of the pressure the way it is applied, there would be less disturbance at the point of contact. But this, of course, could only be done by sacrificing the efficiency of the mechanism. If we had to change the direction of the force it would mean some extra mechanism.

Another instrument has a mechanism for holding it against the tooth to maintain a secure contact of the point and facilitate holding it steady after applying the pressure. It is simply a kind of finger clamp thrown over the tooth in contra direction.

In the college clinic we have used the high pressure method a great deal and with varying success. We keep track of the name and every patient operated on and ask the patient to report in case there is any disturbances in the pulp. So far we have had no adverse reports. We occasionally see articles in the journals stating that this or that operator has had success in almost every case and in just such cases where apparently we have failed. Perhaps it is a lack of skill, or of proper application, or because we do not get our solution just right, or because there is something the matter with the instrument. But to use the humble expression of the farmer: "What will work on one horse will not work on every other horse."

In the general use of cocain for injection the danger of the strong solution is its action on the leucocytes, checking their action and producing emboli, which is most serious when it takes place in the capillary circulation, because then it may produce embuli in the lungs or brain. In an experiment performed on a rabbit the injection of 10 percent failed to kill, because it was injected into the artery, and there it did not have a chance to get into the venous circulation without first passing through the entire circulation, or was taken up by the capillaries before it had a chance to reach the lungs. I think where you can produce an effect at all you can just as well with a weak solution as with a strong solution. I believe that in handling as delicate an organ as the pulp we should use a solution not to exceed 1 or 2 percent; if we can produce an effect at all by careful manipulation, we get it with less danger to the pulp itself.

